# Application of Advanced Machine Learning Approaches to Predict the Compressive Strength of Concrete Containing Supplementary Cementitious Materials

**DOI:** 10.3390/ma14195762

**Published:** 2021-10-02

**Authors:** Waqas Ahmad, Ayaz Ahmad, Krzysztof Adam Ostrowski, Fahid Aslam, Panuwat Joyklad, Paulina Zajdel

**Affiliations:** 1 Department of Civil Engineering, COMSATS University Islamabad, Abbottabad 22060, Pakistan; 2 Faculty of Civil Engineering, Cracow University of Technology, 24 Warszawska Str., 31-155 Cracow, Poland; krzysztof.ostrowski.1@pk.edu.pl (K.A.O.); paulina.zajdel@doktorant.pk.edu.pl (P.Z.); 3 Department of Civil Engineering, College of Engineering in Al-Kharj, Prince Sattam bin Abdulaziz University, Al-Kharj 11942, Saudi Arabia; f.aslam@psau.edu.sa; 4 Department of Civil and Environmental Engineering, Faculty of Engineering, Srinakharinwirot University, 63 Mou 6, Rangsit-Nakhonnayok Rd., Khong 16, Ongkharak District, Nakhon Nayok 26120, Thailand; panuwatj@g.swu.ac.th

**Keywords:** machine learning, concrete, supplementary cementitious materials, fly ash, blast furnace slag, compressive strength

## Abstract

The casting and testing specimens for determining the mechanical properties of concrete is a time-consuming activity. This study employed supervised machine learning techniques, bagging, AdaBoost, gene expression programming, and decision tree to estimate the compressive strength of concrete containing supplementary cementitious materials (fly ash and blast furnace slag). The performance of the models was compared and assessed using the coefficient of determination (R^2^), mean absolute error, mean square error, and root mean square error. The performance of the model was further validated using the k-fold cross-validation approach. Compared to the other employed approaches, the bagging model was more effective in predicting results, with an R^2^ value of 0.92. A sensitivity analysis was also prepared to determine the level of contribution of each parameter utilized to run the models. The use of machine learning (ML) techniques to predict the mechanical properties of concrete will be beneficial to the field of civil engineering because it will save time, effort, and resources. The proposed techniques are efficient to forecast the strength properties of concrete containing supplementary cementitious materials (SCM) and pave the way towards the intelligent design of concrete elements and structures.

## 1. Introduction

Concrete is one of the most frequently used materials in construction worldwide [1,2,3,4,5,6]. The universality of concrete is due to a number of factors, including their ease of acquisition, resistance to water and heat, and adaptability to a variety of sizes and shapes [7,8,9,10,11,12,13,14,15]. However, the production of concrete and its ingredients require a considerable amount of energy, consume natural resources, and release CO_2_ [16,17]. Supplementary cementitious materials (SCMs) are used in concrete to help reduce CO_2_ emissions [18,19,20,21,22,23,24]. Therefore, the utilization of SCMs in concrete could be an effective and sustainable approach. Fly ash (FA) is considered an SCM that could be used as a partial replacement of cement in concrete. Apart from the environmental benefits associated with waste disposal and CO_2_ sequestration [25,26], FA enhances workability, lowers the heat of hydration and thermal cracking at an early age in concrete, and enhances the mechanical performance of concrete, particularly at later ages [27]. Fly ash is formed as a result of the combustion of the hard coal in the temperature range 1250–1400 °C. In terms of chemical composition, the fly ash corresponds to volcanic ash and rocks, such as pumice and trass. FA is a popular additive for the production of concrete, and is treated as a fine-grained non-organic material with pozzolanic properties. The use of FA in fresh concrete mixture improves the concrete mixture’s workability properties and tightness, increases resistance to chemical aggression, and reduces tendencies for draining. FA also delays the mixture binding. As a result of pozzolanic reactions in the long period of time, the durability of concrete with fly ash is higher than the durability of the same concrete made with cement. The delays in binding and concrete hardening help decrease the thermal effects of the hydration of the mixture components. Similarly, blast furnace slag (BFS) can reduce the amount of heat generated during cement hydration and improve the overall performance of concrete, including long-term strength, workability and durability [28]. The BFS is a by-product of pig iron smelting in a blast furnace. Usually, its chemical composition is silicon, aluminum, calcium, magnesium, phosphorus, manganese, sodium, and titanium. It is a material that, when properly crushed and activated, shows binding properties both in the water and in the air. Therefore, it is an essential component of CEM II multi-component Portland cements, CEM III blast furnace cements, and CEM V multi-component cements, and is also used as a type II additive for concrete. When added to concrete, blast furnace slag, similar to fly ash, causes low heat of hydration, higher corrosion resistance, increases the long-term durability of concrete, and improves the concrete mixture’s workability properties. Hence, the utilization of FA and BFS in concrete could help reduce CO_2_ emissions by decreasing the demand for cement and by producing materials with improved mechanical properties.

Supervised machine learning (SML) methods are widely employed in the fields of artificial intelligence and computer science and have an irrefutable impact on engineering. They have quickly achieved popularity in civil engineering, particularly for the prediction of the mechanical strength of materials. SML techniques can be used for prediction results with great accuracy. Ahmad et al. [29] used individual and ensemble machine learning (ML) approaches to foretell the compressive strength (CS) of FA-based concrete. Su et al. [30] compared the accuracy of predictions made using a multi-linear regressor, support vector machine, and artificial neural network to predict the bond strength between fiber-reinforced polymers (FRPs) and concrete. Nguyen et al. [31] forecasted the CS of eco-friendly FA-based geopolymer concrete utilizing ML algorithms. Ahmad et al. [32] predicted the chloride penetration in concrete containing waste material using a decision tree, artificial neural network, and gene expression programming techniques. Gene expression programming was demonstrated to be a more effective prediction technique than other algorithms. However, further exploration is required in this area to better understand and apply these approaches in civil engineering while utilizing different datasets and SML techniques.

Considerable experimental research has been carried out to determine the mechanical properties of SCM-based concrete. However, casting and testing specimens in the laboratory require time, effort, and cost. This study concentrates on applying SML techniques for forecasting the CS of concrete containing FA and BFS. The bagging, Adaptive Boosting (AdaBoost), gene expression programming (GEP), and decision tree (DT) algorithms were used to predict the CS of concrete. Numerous statistical checks, error distribution, and the k-fold cross-validation approaches were involved to ensure that the models perform as expected. The purpose of this research was to apply SML algorithms (bagging and AdaBoost) to predict the CS of 1030 data points of concrete containing FA and BFS. Additionally, this research aimed to compare the performance of bagging and AdaBoost techniques using the coefficient of determination (R^2^) value. To assess in the implementation of both models, statistical checks, error evaluation, including mean absolute error (MAE), mean square error (MSE), and root mean square error (RMSE), k-fold cross-validation, and sensitivity analysis were used. This research could be beneficial for scholars in civil engineering because it allows for the prediction of strength properties without requiring precious time in the laboratory.

## 2. Research Methodology

### 2.1. Data Description

**Figure 1:**
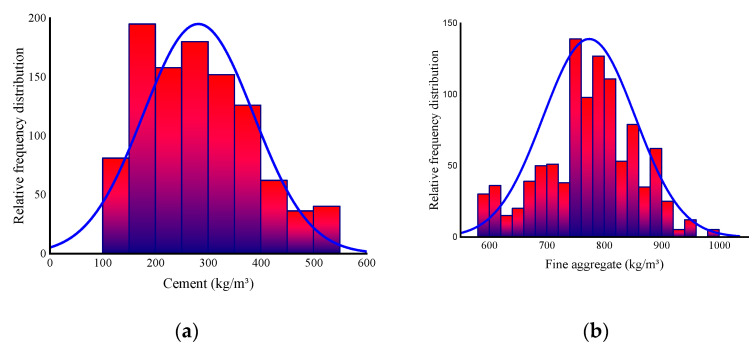

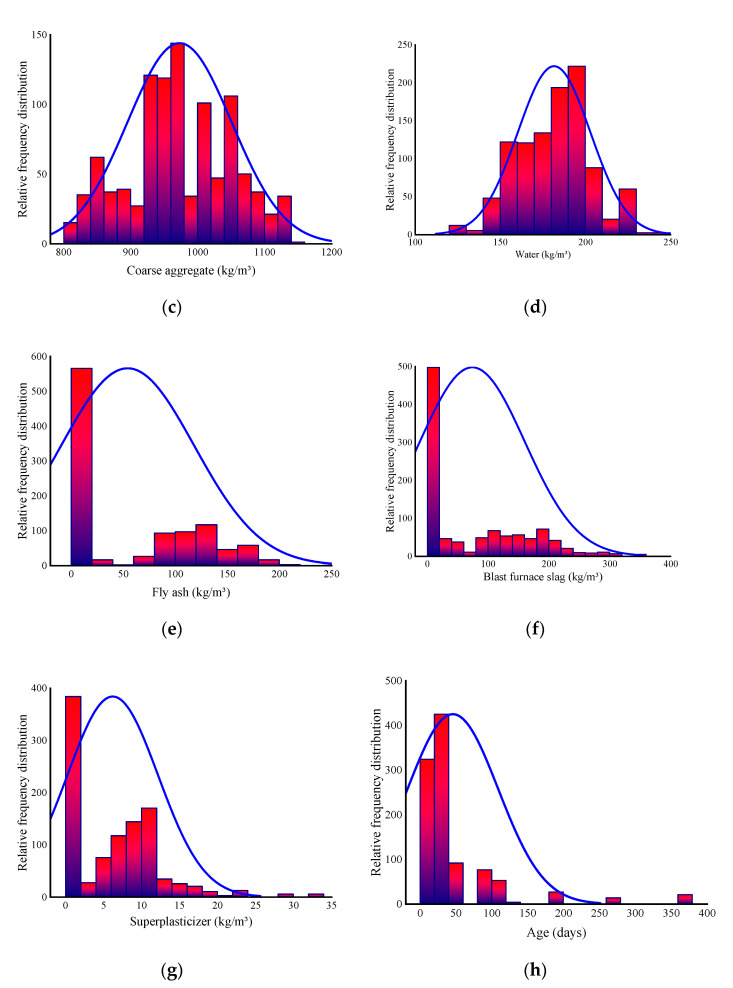
Relative frequency distribution of input parameters: (**a**) Cement; (**b**) Fine aggregate; (**c**) Coarse aggregate; (**d**) Water; (**e**) Fly ash; (**f**) Blast furnace slag; (**g**) Superplasticizer; (**h**) Age.

To produce the predicted output variable, SML algorithms need various input variables [33,34]. The data utilized in this work to forecast the CS of concrete using FA and BFS as SCM were derived from the literature [35,36,37,38]. A total of eight factors were used as inputs for the models, including cement, FA, BFS, water, superplasticizer, coarse aggregate, fine aggregate, and age, while one variable, CS, was used as an output. The input variables and the number of data points have a significant impact on the model’s output [29,32,39]. The study used a total of 1030 data points (mixes) for the CS prediction of concrete incorporating FA and BFS. Table 1 shows the descriptive statistical analysis for all the input parameters. Figure 1 shows the relative frequency distribution of each parameter utilized in the mixes.

### 2.2. Techniques Employed

Anaconda software was employed to execute the bagging, AdaBoost, and DT models using Python code. The Anaconda navigator is a desktop graphical user interface incorporate in the Anaconda software, which allows for launching applications that can direct Conda packages, environments, and channels without the need to employ command-line commands. It is also a distribution point of Python and R programming languages for data science and ML applications that focus on clarifying package development and management. In addition, GEP software was employed to run the GEP model. To estimate the CS of SCM-based concrete, the study used four techniques, namely Bagging, AdaBoost, GEP, and DT. Spyder (version: 4.3.5) was chosen from the Anaconda navigator for running the models. The coefficient of determination (R^2^) value of the expected outcome from all models indicated the degree of accuracy. The R^2^ values typically range between 0 and 1, with a larger number indicating more accuracy between the actual and projected results. The ensemble technique is an ML concept that is used to train several models using a similar learning algorithm [40]. The ensemble consists of a large number of algorithms collectively referred to as multi-classifiers. A group of hundreds or thousands of learners with the same goal gets together to resolve the issue.

### 2.3. Description of Machine Learning Algorithms

Bagging is an ensemble method of the parallel kind that explains the variance of the prediction model by providing supplementary data during the training stage. This production is based on irregular sampling, which includes data substitution from the real set. Certain observations can be replicated in each new training data set using sampling with replacement. Each component has an equal chance of appearing in the new dataset in the bagging technique. Increases in the size of the training set have no effect on the predictive force. Additionally, the deviation can be significantly decreased by fine-tuning the forecast to an anticipated result. All these data sets are often utilized for training additional models. This ensemble of various models uses the mean of all the forecasts made by the various models. The prediction in regression can be the mean or average of the predictions from several models [41].

AdaBoost is an SML approach that makes use of ensemble learning. It is also known as adaptive boosting since the weights are re-given to each instance, with increased weights applied to instances that were incorrectly categorized. Boosting techniques are commonly employed in SML to reduce bias and variation. These ensemble approaches are intended to strengthen the weak learner. During the training phase for the input data, an infinite number of DTs are utilized. While constructing the first DT/model, a significant priority is placed on the recorded data that is incorrectly classified throughout the initial model. These data records are the only ones that are utilized as input for another model. The procedure outlined above will be continued until the desired number of basic learners is obtained. On binary classification problems, the AdaBoost regressor outperforms all other regressors in terms of improving the performance of DTs. Furthermore, it is utilized to improve the performance of other machine learning methods. The AdaBoost algorithm provides several advantages when it comes to handling multiple mapping problems. Gradual learning is used to assign different weights to weak classifiers with poor accuracy, and each weak classifier’s weight is completely evaluated to produce a high-precision strong classifier [42,43].

Decision tree (DT) is a supervised ML method that is used to solve regression and classification issues. The DT is a tool that visualizes a tree-like model of decisions and their feasible outcomes. The DT’s structure is like that of a flowchart, with nodes, branches, and roots. Each internal node displays a test on an attribute, each branch displays the result of the test, and each leaf node displays the class tag. The categorization rule is represented by the path that the root takes to reach the leaf. Three distinct types of DT nodes are accessible, each with a distinct geometric shape (square, circle, and triangle). It is a rather straightforward strategy to comprehend and interpret.

Gene expression programming (GEP) is a transformational algorithm for creating computer programs and modules. These programmers typically contain a tree structure that can change its size (dimensions, shape, and layout), as chromosomes do. As a result, GEP, as a genotype-phenotypic system, can be far more effective than adaptive approaches. GEP’s programming language is called Karva and is identical to the LISP languages. GEP has numerous advantages over other classical regression approaches, as other techniques create some functions first and then analyze them. However, no preset function is considered in GEP. In this study, statistical checks, error evaluation, including MAE, MSE, RMSE, K-fold cross-validation, and sensitivity analysis were performed to assess the performance of all the employed models.

**Figure 2:**
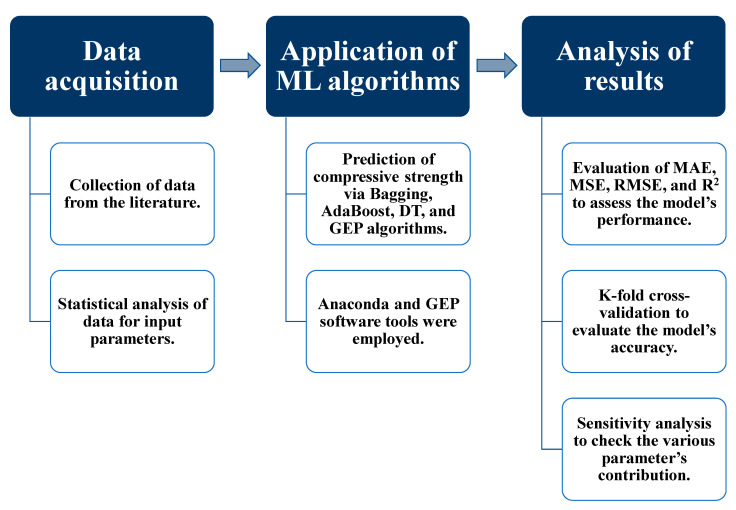
Research methodology flow chart.

Figure 2 depicts the research approach as a flowchart.

## 3. Results and Analysis

### 3.1. Statistical Analysis

**Figure 3:**
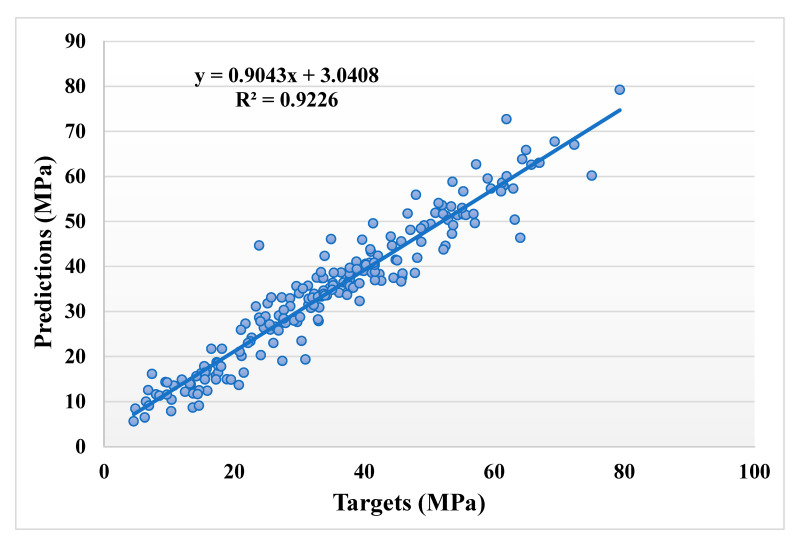
Relationship between the actual and predicted results of the bagging model.

**Figure 4:**
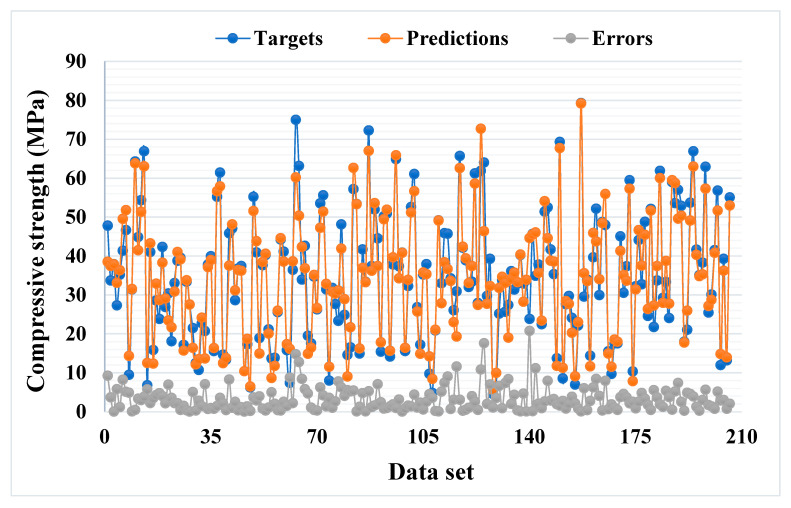
Distribution of actual and predicted values along with the errors for the bagging model.

Figure 3 illustrates the statistical analysis interpretation of the real and anticipated results for the CS of SCM-based concrete for the bagging model. The bagging produces output with great precision and a low divergence amongst the real and anticipated values. The R^2^ value of 0.92 indicates the model’s higher accuracy in terms of results prediction. Figure 4 illustrates the scattering of experimental values (targets), predicted values, and errors for the bagging model. The distribution’s highest, lowest, and average values were 20.79, 0.004, and 3.26 MPa, respectively. However, 26.21% of the error data were less than 1 MPa, whereas 51.94% of the error data were between 1 and 5 MPa, 18.45% were between 5 and 10 MPa, and only 3.40% of the error data were greater than 10 MPa.

**Figure 5:**
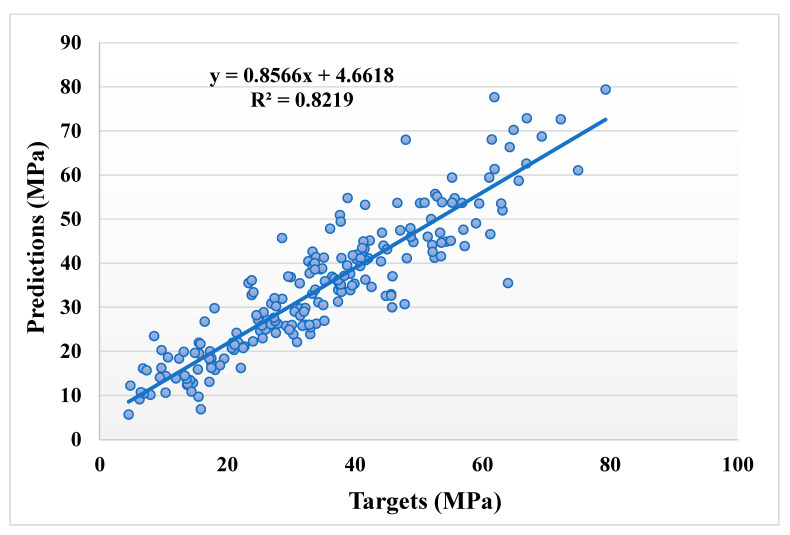
Relationship between the actual and predicted results of the AdaBoost model.

**Figure 6:**
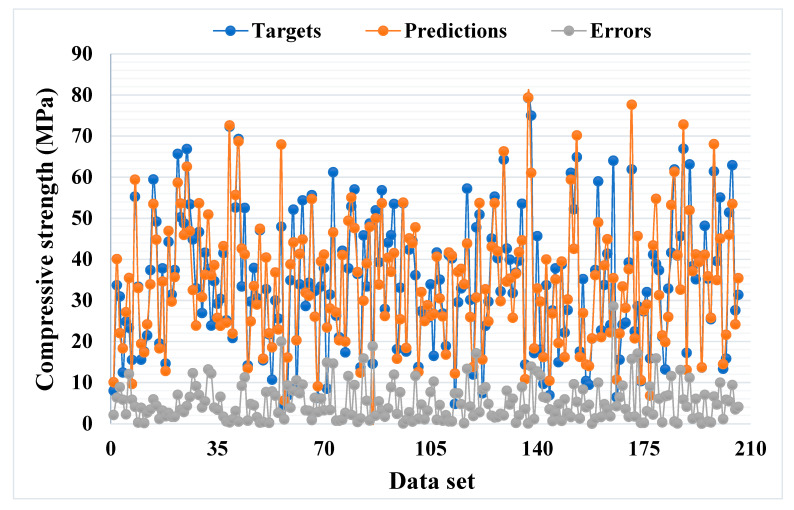
Distribution of actual and predicted values along with the errors for the AdaBoost model.

Figure 5 and Figure 6 illustrate a comparable comparison of actual and anticipated outcomes for the AdaBoost model. The correlation between the actual and projected results is depicted in Figure 5, with an R^2^ value of 0.82, which is within an acceptable range with less variation than the bagging model. The distribution of actual values (targets), predicted values, and errors for the AdaBoost model is depicted in Figure 6. The distribution’s highest, lowest, and average values were 28.57, 0.020, and 5.13 MPa, respectively. However, 16.99% of the error values were less than 1 MPa, while 43.20% were between 1 and 5 MPa, 26.70% were between 5 and 10 MPa, and only 13.11% were larger than 10 MPa. The comparison between bagging and AdaBoost models (R^2^ and error distribution) indicated that the bagging model can more accurately predict the CS of SCM-based concrete. Furthermore, twenty sub-models were employed in both techniques to identify the optimal value that results in an uncompromising output result.

**Figure 7:**
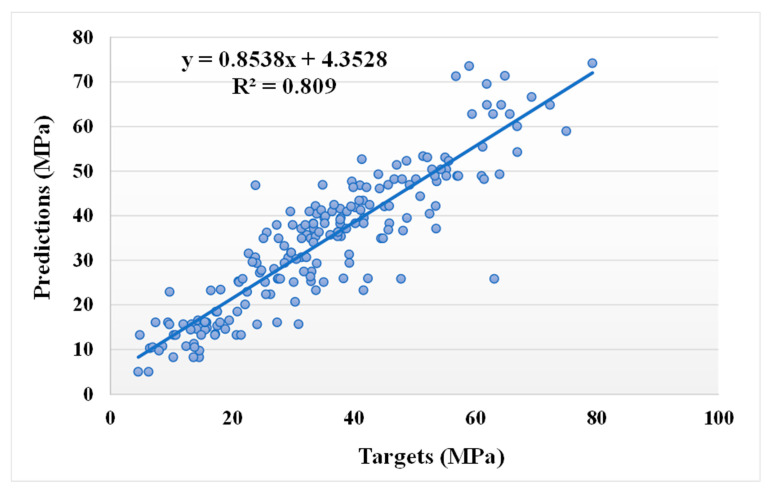
Relationship between the actual and predicted results of the GEP model.

**Figure 8:**
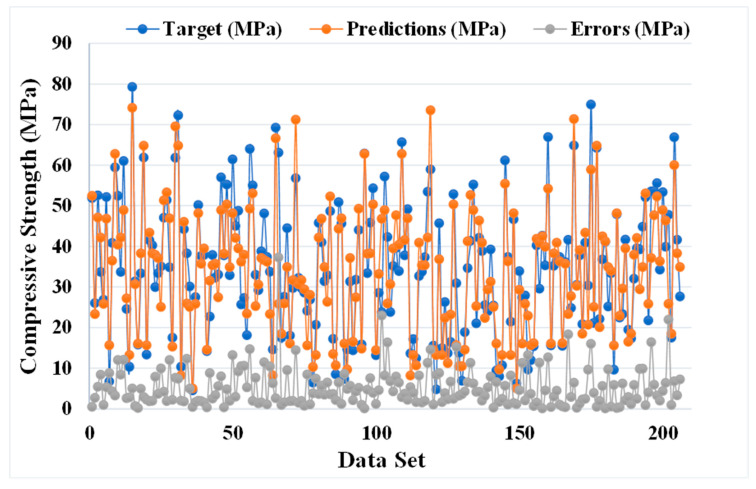
Distribution of actual and predicted values along with the errors for the GEP model.

Figure 7 and Figure 8 show the comparison between the actual and anticipated outcomes for the GEP model. The correlation between the targeted and projected outcome is shown in Figure 7, indicating the value of R^2^ equals 0.81, which is also within an acceptable range with less variation. The distribution of actual values (targets), predicted values, and errors for the GEP model can be seen in Figure 8. The error distribution’s highest, lowest, and average values were 37.30, 0.11, and 5.24 MPa, respectively. We noted that 11.16% of the error values were between 0 and 1 MPa, 48.54% of the error values were between 1 and 5 MPa, 27.67% of the error values were between 5 and 10 MPa, and only 12.62% of the error values were above 10 MPa. In comparison to the bagging and AdaBoost models, the GEP model’s accuracy is less in terms of predicting the CS of SCM-based concrete, as indicated by the R^2^ value.

**Figure 9:**
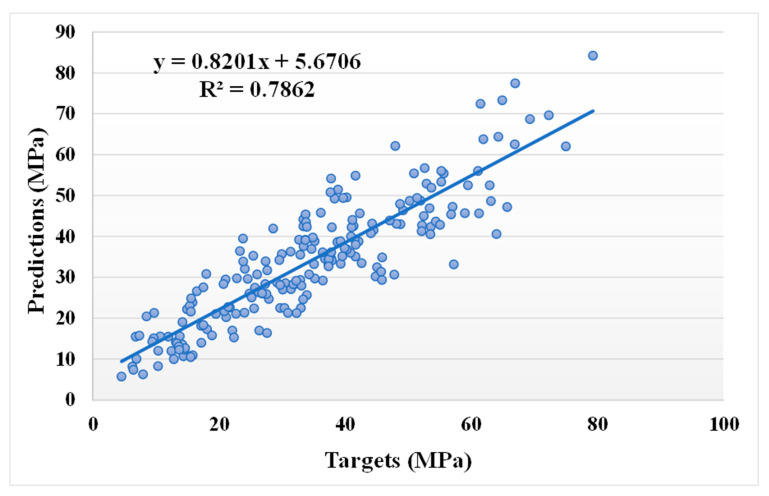
Relationship between the actual and predicted results of the DT model.

**Figure 10:**
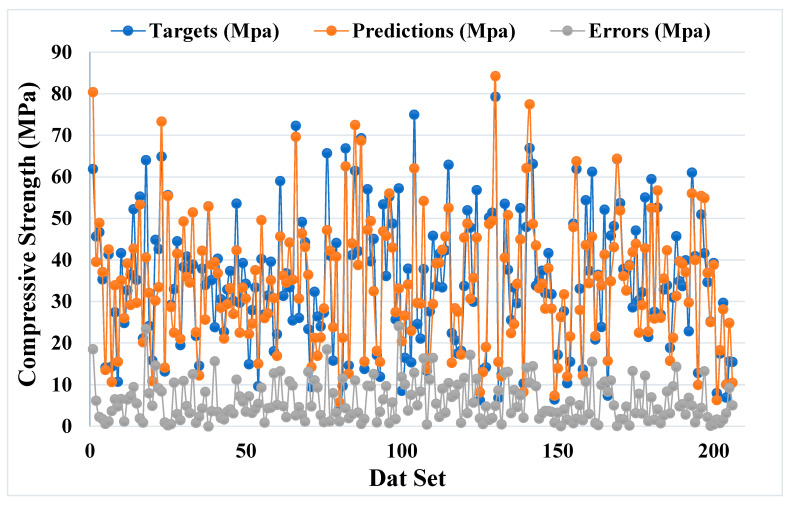
Distribution of actual and predicted values along with the errors for the DT model.

Figure 9 indicates the statistical analysis interpretation of the actual and forecasted results for the CS of SCM-based concrete for the DT model. The DT also gives the output with lesser accuracy between the targeted and anticipated results. DT models give an R^2^ value of 0.79, which indicates the lowest accuracy of the model as compared to the other employed ML approaches. Figure 10 illustrates the scattering of experimental values (targets), predicted values, and errors for the bagging model. We noted that the error distribution’s highest, lowest, and average values were 24.05, 0.01, and 5.88 MPa, respectively. We observed that 13.11% of the error values were between 0 and 1 MPa, 41.75% of the error values were between 1 and 5 MPa, 17.96% of the error values were between 5 and 10 MPa, and 20.87% of the error values were above 10 MPa. In comparison, the ensemble ML approaches (bagging and AdaBoost) are more effective for predicting the CS of SCM-based concrete as opposed to the individual ML techniques (GEP and DT), as indicated by their R^2^ values.

### 3.2. K-Fold Cross-Validation

The k-fold cross-validation approach was used to determine the model’s legitimacy during execution. The k-fold cross-validation procedure is typically used to determine the model’s validity [33], in which the relevant data are randomly distributed and split into 10 groups. Nine groups are designated for training, and one is designated for validating the model. From the data, 80% was allocated to train the models, while the remaining 20% was assigned to test the employed models. The lesser value of the errors (MAE, MSE, RMSE) and the high value of the R^2^ indicate the model’s accuracy. Additionally, the technique needs to be repeated 10 times to achieve an average outcome. This thorough process leads to the model’s excellent precision. Furthermore, statistical assessment in the form of errors (MSE, MAE, and RMSE) was performed, as demonstrated in Table 2. The models’ reaction to prediction was evaluated through applying statistical analysis using the following Equations (1)–(3), retrieved from the literature [44,45]:

(1) $$\mathrm{MAE}=\frac{1}{n}\overset{n}{\underset{i=1}{\sum }}\left|x_{i}-x\right|$$

(2) $$\mathrm{MSE}=\frac{1}{n}\overset{n}{\underset{i=1}{\sum }}{\left(y_{pred}-y_{ref}\right)}^{2}$$

(3) $$\mathrm{RMSE}=\sqrt{\sum^{\;}\frac{{\left(y_{pred}-y_{ref}\right)}^{2}}{n}}$$

where *n* = total number of data samples, x, $y_{ref}$ = reference values in the data sample, and $x_{i}$, $y_{pred}$ = predicted values from models.

R^2^, MAE, MSE, and RMSE were used to evaluate the k-fold cross-validation, and their distributions for bagging, AdaBoost, GEP, and DT models are shown in Table 3. The bagging model with the lowest error and a high R^2^ value has a better performance in terms of results prediction. The highest, lowest, and average R^2^ values for the bagging model were 0.92, 0.58, and 0.77, respectively. Comparably, the highest, lowest, and average R^2^ values for the AdaBoost model were 0.86, 0.40, and 0.68, respectively. Moreover, the MAE, MSE, and RMSE maximum values for the bagging model were 6.44, 93.27, and 9.66 MPa, respectively, whereas the same values for the AdaBoost model were 11.37, 352.19, and 18.77 MPa. The bagging model’s minimal MAE, MSE, and RMSE were 3.80, 23.84, and 4.88 MPa, respectively, whereas the same value for the AdaBoost model was 5.78, 55.82, and 7.47 MPa. Similar values for GEP and DT models are also listed in Table 3.

### 3.3. Sensitivity Analysis

**Figure 11:**
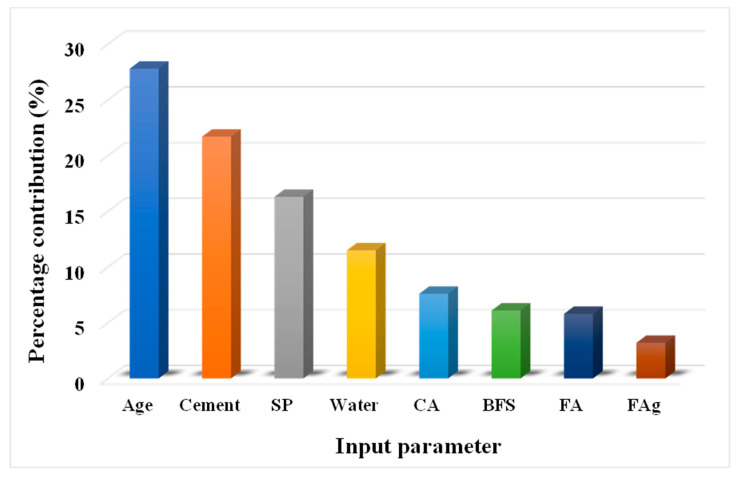
Sensitivity analysis of various contributing parameters towards the prediction. SP: superplasticizer, CA: coarse aggregate, BFS: blast furnace slag, FA: fly ash, FAg: fine aggregate.

This investigation is concerned with the influence of input parameters on forecasting the CS of SCM-based concrete. The input parameters have a considerable impact on the outcome projection [46]. The influence of each input parameter on the CS prediction of concrete is depicted in Figure 11. We noted that age contributed the most (27.8%), while cement and superplasticizer contributed 21.7% and 16.3%, respectively. However, the other variables contributed less, with water contributing 11.5%, coarse aggregate 7.6%, BFS 6.1%, FA 5.8%, and fine aggregate 2.1% to the prediction of CS of SCM-based concrete. The sensitivity analyses produced results proportional to the number of input parameters and data points utilized to run the model. However, the applied ML method identified the influence of each parameter, and this analysis produced varying results due to the variation in concrete mix proportions and the addition of other input parameters. The following Equations (4) and (5) were used to determine each variable’s contribution to the model’s output:

(4) $$N_{i}=f_{max}\left(x_{i}\right)-f_{min}\left(x_{i}\right)$$

(5) $$S_{i}=\frac{N_{i}}{\sum_{j-i}^{n}N_{j}}$$

where, $f_{max}\left(x_{i}\right)$ and $f_{min}\left(x_{i}\right)$ are the highest and lowest of the anticipated output over the ith output.

## 4. Discussions

**Figure 12:**
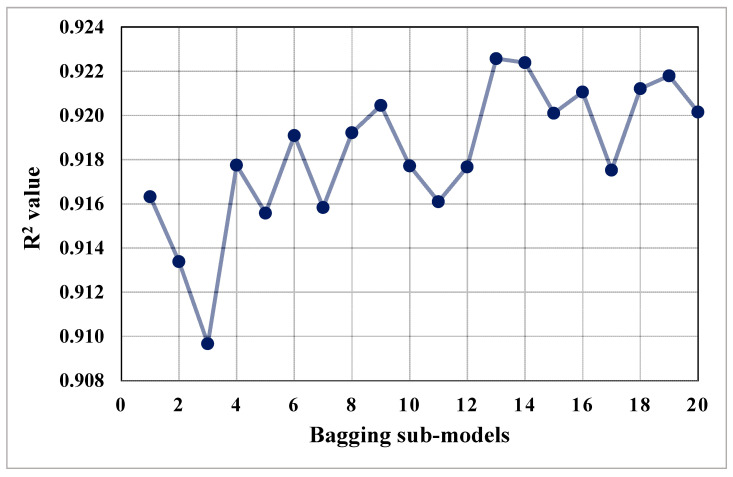
Coefficient of determination (R^2^) values of bagging sub-models.

**Figure 13:**
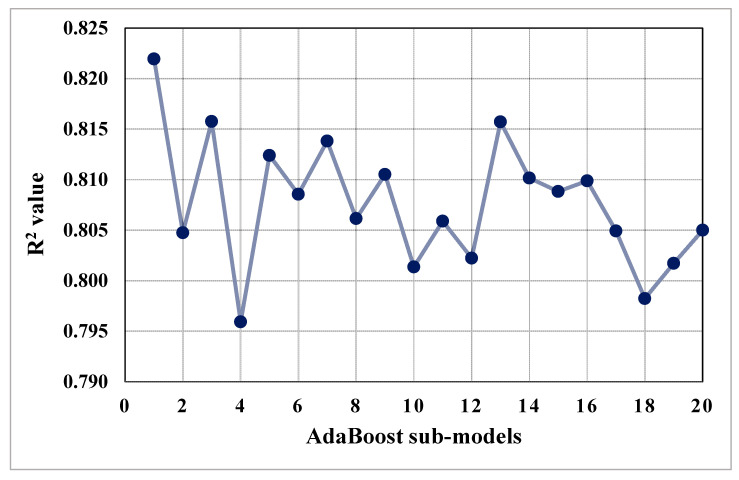
Coefficient of determination (R^2^) values of AdaBoost sub-models.

The purpose of this study was to demonstrate how SML techniques may be used to predict the CS of concrete incorporating FA and BFS as cement replacements. The purpose of using SCMs in concrete is to create material that is both effective and sustainable. The study employed four ML techniques, namely bagging, AdaBoost, GEP, and DT. To determine which algorithm was the better predictor, the forecast performance of all the employed techniques was contrasted. The bagging model’s output was more accurate, exhibiting an R^2^ value of 0.92, compared to the AdaBoost, GEP, and DT models indicating R^2^ values equal to 0.82, 0.81, and 0.79, respectively. Additionally, the performance of all the models was validated using statistical assessment and the k-fold cross-validation approach. The lower the error levels, the higher performing the model is. However, analyzing and recommending the optimum ML regressor for predicting results for diverse topics is challenging because each model’s performance is strongly related to the input parameters and data points used to run the model. However, ensemble ML techniques typically employ the weak learner by constructing sub-models that can be trained on data and optimizing to reach the largest R^2^ value. The distribution of R^2^ values of sub-models for bagging and AdaBoost techniques is depicted in Figure 12 and Figure 13, respectively. We observed that the R^2^ values for all sub-models of bagging were higher than 0.91 (Figure 12), while the maximum R^2^ value among all the sub-models of AdaBoost was 0.82 (Figure 13). This also supports the higher accuracy of the bagging technique for the prediction of the results. The literature also indicated that bagging models produce more accurate outcomes than other ML approaches [29,46]. Moreover, a sensitivity analysis was performed to determine the impact of each input parameter on the prediction of the CS of SCM-based concrete. The model’s performance could be influenced by the input parameters and the amount of data points. The contribution level of each of the eight input parameters to the anticipated result was determined by the sensitivity analysis. 

## 5. Conclusions

The objective of this study was to evaluate and demonstrate how supervised machine learning (SML) techniques can be used to forecast the compressive strength of concrete incorporating fly ash and blast furnace slag. The CS of concrete was forecasted using bagging, Adaptive Boosting (AdaBoost), gene expression programming (GEP), and decision tree (DT) algorithms. The following conclusions can be drawn:

- The bagging model was more effective at prediction than the other employed models, as evidenced by its better coefficient of determination (R^2^) and lower error values. The R^2^ for the bagging, AdaBoost, GEP, and DT models was noted to be 0.92, 0.82, 0.81, and 0.79, respectively. However, the results of all the models were in the acceptable range.
- Statistical analysis and the k-fold cross-validation method have also proven the satisfactory performance of the employed models. These checks also corroborated the bagging model’s superior performance over the other analyzed models.
- Sensitivity analysis of input parameters revealed that coarse aggregate, fine aggregate, and cement contributed 24.7%, 18.4%, and 16.2%, respectively, to the prediction of the results, while other input parameters contributed less.
- The SML techniques can forecast the strength properties of concrete with higher accuracy without requiring additional time for sample casting and testing.

The use of both the ensemble (bagging and AdaBoost) and individual (GEP and DT) ML techniques to forecast concrete CS was proposed in this work. It is recommended that other SML techniques be employed to compare their accuracy for the prediction of outcomes. Moreover, data points and the number of outcomes should be expanded through experimental work, field tests, and other numerical analyses utilizing other methodologies (e.g., Monte Carlo simulation) in future studies. Moreover, the input parameters could be enhanced by adding environmental factors (e.g., high temperature and humidity), along with the in-depth explanation of the raw materials to get a better response from the models. In addition, more in-depth analyses, checks, and effects should be incorporated for a better evaluation and understanding of the outcomes from ML techniques.

## Figures and Tables

**Table 1:** Descriptive analysis of input parameters.

Parameter	Input Variable							
	Cement [kg/m^3^]	Blast Furnace Slag [kg/m^3^]	Fly Ash [kg/m^3^]	Water [kg/m^3^]	Superplasticizer [kg/m^3^]	Coarse Aggregate [kg/m^3^]	Fine Aggregate [kg/m^3^]	Age [days]
Mean	281.17	73.90	54.19	181.57	6.20	972.92	773.58	45.66
Standard Error	3.26	2.69	1.99	0.67	0.19	2.42	2.50	1.97
Median	272.90	22.00	0.00	185.00	6.35	968.00	779.51	28.00
Mode	425.00	0.00	0.00	192.00	0.00	932.00	594.00	28.00
Standard Deviation	104.51	86.28	64.00	21.36	5.97	77.75	80.18	63.17
Range	438.00	359.40	200.10	125.25	32.20	344.00	398.60	364.00
Minimum	102.00	0.00	0.00	121.75	0.00	801.00	594.00	1.00
Maximum	540.00	359.40	200.10	247.00	32.20	1145.00	992.60	365.00

**Table 2:** Statistical checks of the models.

Machine Learning Technique	MAE (MPa)	MSE (MPa)	RMSE (MPa)
Bagging	3.257	20.566	4.535
AdaBoost	5.126	47.376	6.883
Gene expression programming	5.24	50.69	7.12
Decision tree	5.88	57.30	7.57

**Table 3:** Results of k-fold cross-validation.

K-Fold	Bagging				AdaBoost				GEP				DT			
	MAE	MSE	RMSE	R^2^	MAE	MSE	RMSE	R^2^	MAE	MSE	RMSE	R^2^	MAE	MSE	RMSE	R^2^
1	6.32	93.27	9.66	0.78	11.37	352.19	18.77	0.79	8.82	144.16	12.01	0.64	7.19	104.48	10.22	0.79
2	3.8	23.84	4.88	0.89	6.98	88.87	9.43	0.66	4.96	44.64	6.68	0.81	7.06	89.92	9.48	0.88
3	4.02	57.12	7.56	0.75	7.14	87.44	9.35	0.82	6.97	78.53	8.86	0.69	5.21	50.22	7.09	0.64
4	6.21	59.64	7.72	0.68	8.52	120.48	10.98	0.55	7.82	107.62	10.37	0.97	7.79	108.75	10.43	0.98
5	6.44	53.77	7.33	0.76	5.78	69.19	8.32	0.47	6.78	68.60	8.28	0.94	6.00	70.30	8.38	0.91
6	5.25	80.87	8.99	0.78	10.45	305.18	17.47	0.83	8.11	91.29	9.55	0.88	8.39	130.51	11.42	0.71
7	4.27	26.34	5.13	0.92	6.6	55.82	7.47	0.86	5.18	44.09	6.64	0.72	5.38	53.56	7.32	0.84
8	4.72	54.81	7.4	0.58	6.68	83.73	9.15	0.4	8.55	129.71	11.39	0.76	7.91	100.65	10.03	0.93
9	5.68	55	7.42	0.73	7.33	92.23	9.6	0.68	9.25	154.31	12.42	0.95	5.26	48.27	6.95	0.66
10	4.57	47.7	6.91	0.83	5.89	56.63	7.53	0.74	6.82	79.24	8.90	0.93	5.25	58.76	7.67	0.75

## Data Availability

Not applicable.
